# Pivotal Role of Ultrasonography and Radiology in Diagnosing a Case of Sialolith

**DOI:** 10.7759/cureus.51269

**Published:** 2023-12-29

**Authors:** Ashish Lanjekar, Monal M Kukde, Isha Madne, Komal R Deotale, Nandkishor J Bankar

**Affiliations:** 1 Oral Medicine and Radiology, Swargiya Dadasaheb Kalmegh Smruti Dental College and Hospital, Nagpur, IND; 2 Dentistry, Datta Meghe Medical College, Datta Meghe Institute of Medical Sciences, Nagpur, IND; 3 Microbiology, Jawarhal Nehru Medical College, Datta Meghe Institute of Medical Sciences, Wardha, IND

**Keywords:** salivary glands, ultrasonography, submandibular gland, sialolithiasis, sialolith

## Abstract

Sialolithiasis is a condition that affects the salivary glands, which commonly occurs within the body of the submandibular gland or the Wharton duct. This condition is characterised by pain in the submandibular area after meals. Conservative therapies such as duct milking and palliative care can provide positive results for small, easily accessible calculi. This report describes the results of radiographic imaging of a 43-year-old patient with pain and swelling in the submandibular region. During the extraoral examination, a 1.5 cm wide diffuse swelling was present in the left submandibular region, and the left submandibular gland was tender and firm. In addition, a solitary, tender left submandibular lymph node was observed. Intraorally, the opening of the submandibular duct was erythematous and inflamed. The patient was advised for necessary investigations, including an orthopantomogram, cone beam CT, neck ultrasound, and left submandibular gland sialography. Ultrasonography was preferred over other imaging techniques due to its non-invasive nature and high accuracy, sensitivity, and specificity in diagnosing sialolithiasis. Timely management of sialolith is critical as delayed treatment can lead to serious consequences. A conclusive diagnosis of left submandibular sialolithiasis, accompanied by sialadenitis, was made based on clinical, radiographic, and ultrasound findings.

## Introduction

Sialolithiasis is the formation of stones or calculi in the salivary gland or duct. This disorder is reported to affect one in 10,000 people and is considered one of the primary causes of enlarged salivary glands [1]. Sialoliths can potentially obstruct the salivary ducts, leading to inflammation, sialadenitis, or the formation of abscesses. The primary indications of this condition include reduced salivary flow and periodic post-meal swelling of the affected gland [2]. Sialoliths are more commonly found in the submandibular gland, which contains 80-95% of them, compared to the parotid gland, which only has 5%-20%. Intraductal localized salivary calculi are more prevalent than intraglandular calculi [3]. The submandibular gland is more likely to produce sialoliths due to its anatomical position, its longer and more convoluted canal, and the generation of mucin-rich alkaline saliva, which makes it more difficult for the saliva to flow against gravity. Most submandibular calculi are detected on sialography as radiolucent filling deficiencies and on plain radiographs as radiopaque masses. For 20% of sialoliths that are not radiopaque, a sialography or sialendoscopy may be required for diagnosis. Unilateral salivary calculi, for the most part, do not produce dry mouth [4]. This is a case report of submandibular sialolithiasis, with detailed radiological and sialographic findings. It underlines the critical importance of timely management of sialolith, demonstrating the potentially serious consequences of delayed treatment. 

## Case presentation

**Figure 1:**
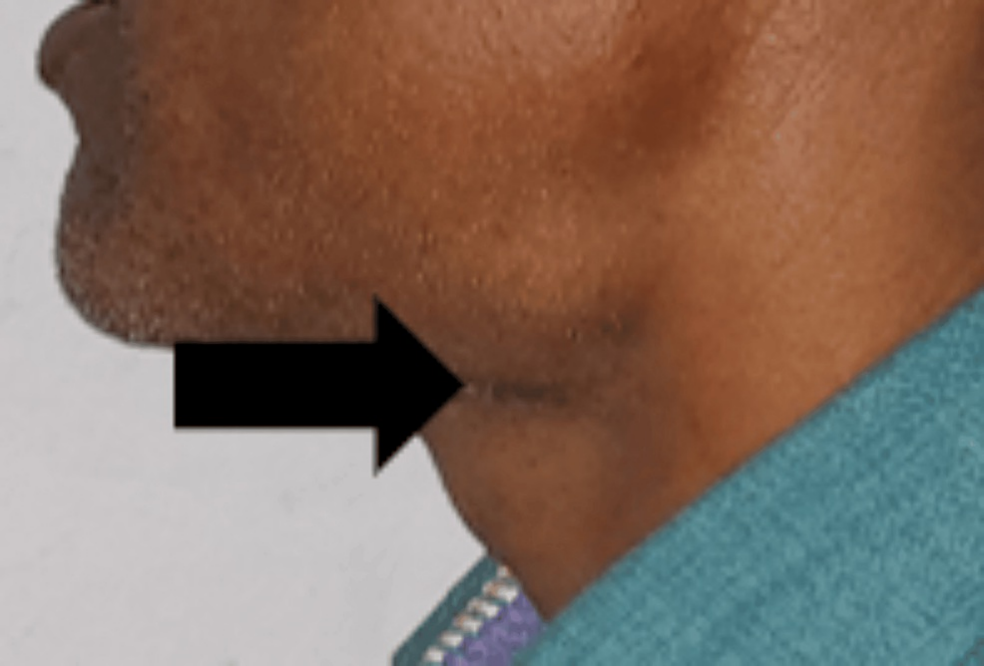
Extraoral swelling in the left submandibular region

A 43-year-old male patient reported to the department with a medical history of recurring episodes of pain, dysphagia, and neck swelling that persisted for a year. The patient had experienced a similar episode approximately two months before the scheduled appointment, and the pain had persisted since then. An extraoral inspection revealed a 1.5 cm wide diffuse swelling in the left submandibular region. Bimanual palpation confirmed the presence of a tender and firm left submandibular gland and a solitary tender left submandibular lymph node (Figure 1).

**Figure 2:**
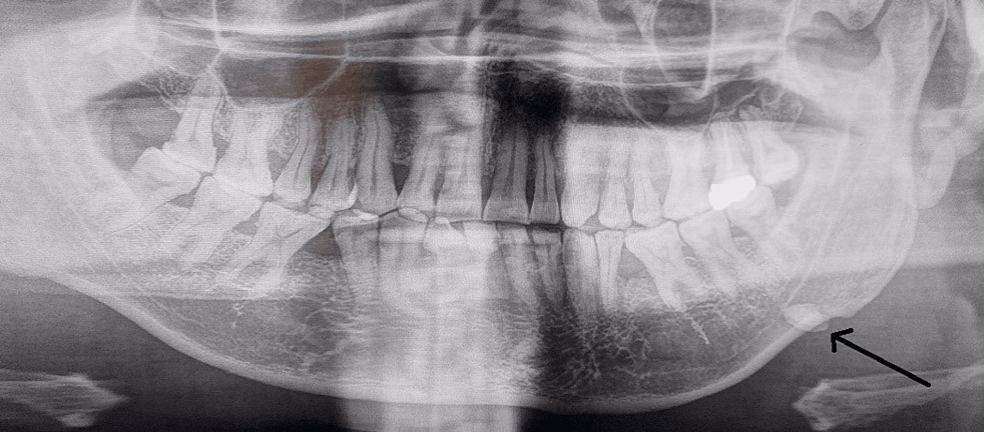
Orthopantomogram showing ovoid calcification along the lower border of the mandible on the left side

**Figure 3:**
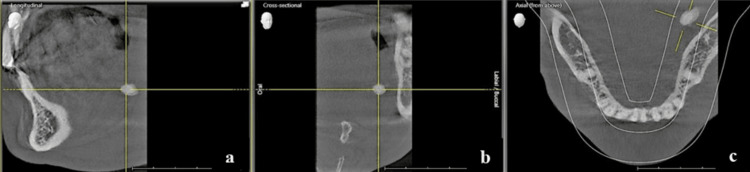
Longitudinal, cross-sectional, and axial view of cone beam CT showing sialolith along the medial aspect of the mandible Longitudinal section (a), cross-section (b), and axial section (c) showing radiopaque sialolith in crosshair along the medial aspect of the mandible.

The opening of the submandibular duct was found to be inflamed, but there was no indication of swelling present in the oral cavity. Orthopantomogram revealed a single ovoid radiopacity located along the lower border of the mandible, extending from the distal tip of tooth 37 to the mesial of tooth 38 (Figure 2). Additionally, radiopacity was detected in the medial region of the mandible body on the axial section of the cone beam CT (CBCT) (Figure 3).

The patient was also advised for an ultrasound neck, which revealed that the left submandibular gland was enlarged, with altered echotexture and lobulated borders, measuring around 4.7 * 2.5 cm. It also revealed 11 mm of calcification within it. On colour Doppler, there was very little vascularity. A few swollen lymph nodes at Level II were also found.

**Figure 4:**
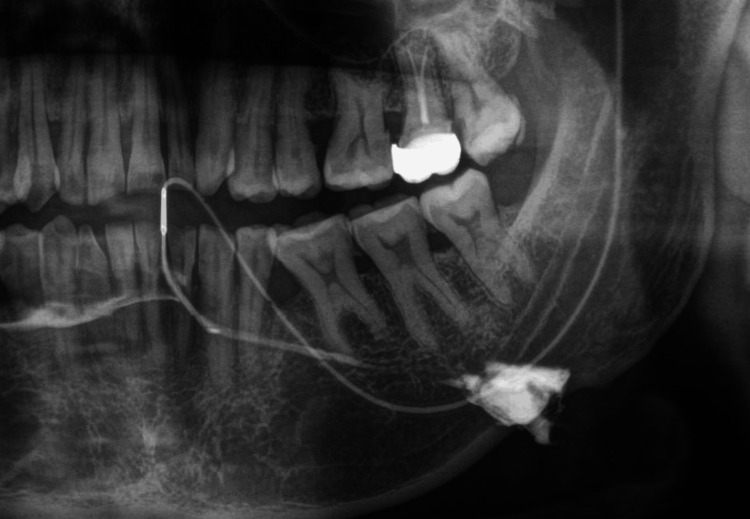
Orthopantomogram showing accumulation of water-soluble non-ionic contrast media in association with sialolith

The left submandibular gland sialography was performed with water-soluble nonionic iodine contrast media that confirmed the presence of sialolith within the gland (Figure 4).

Based on clinical, radiographic, and ultrasonographic evidence, the case was diagnosed as left submandibular sialolithiasis with sialadenitis. The patient was advised to undergo gland removal surgery, but the patient did not show up for the surgery.

## Discussion

Sialoliths are believed to have originated due to mineral salt deposition around an original nidus composed of bacteria, salivary mucins, or desquamated epithelial cells, while the exact source is unknown [4]. A decrease in salivary flow, an increase in alkalinity, and increased calcium levels can all have an impact on the development of sialoliths. Due to the direction of salivary flow against gravity, a longer and more complex duct structure, and a higher amount of calcium and mucus in the saliva produced by the submandibular gland, it is more vulnerable to salivary gland calculi than the parotid gland [5].

Often sialolith has a maximum diameter of 5 mm and any stone larger than 10 mm is considered significant-sized sialolith. In this particular case, the sialolith measured 11 mm in length, which is an unusual finding [3]. Males are twice as likely to be affected [6]. The two stages of sialolith production are described in the literature as (a) core formation in the centre and (b) creation of surrounding layers. The central core is created by the precipitation of mineral salts bound by organic molecules. Several inorganic and organic components then encircle the central nucleus. According to theory, calculi typically form in the submandibular and parotid glands when there is, respectively, a nidus of mucus and a central core of inflammatory cells or a foreign body. For the accurate diagnosis and treatment of these glandular stones, it is essential to recognize the contributing causes [7]. Sialolithiasis is usually asymptomatic; however, the accumulation of saliva can cause pain and swelling in the afflicted gland if a salivary calculus blocks the Wharton's duct lumen. Furthermore, bacterial invasion of the gland parenchyma can result in recurrent infections. In the present case, the patient reported similar symptoms of having pain after taking meals, which suggested the accumulation of saliva in the gland. 

Clinical symptoms and radiography have traditionally diagnosed sialolithiasis or the presence of salivary gland stones. However, advances in diagnostic modalities, such as CT and MRI, have revolutionized the diagnostic process. The development of the sial endoscope has also greatly improved pathology diagnoses. Technological advances have enhanced optical resolution and reduced the size of the device, allowing the detection of radiolucent salivary gland calculi and obstructions that may not be apparent using traditional diagnostic imaging. In addition, sialendoscopy can identify potential duct stricture or other obstructions. In certain circumstances where there is a small sialolith and favourable conditions exist, sialendoscopy may be used to perform a definitive sialolithotomy [8].

In the management of sialolithiasis, conservative procedures, such as instrumental papilla dilation during or after gland stimulation, are generally recommended. Antibacterial drugs are crucial in targeting harmful bacteria and preventing their spread. In order to effectively combat bacterial infections, it is imperative that these drugs are directed against both the Gram-positive and Gram-negative spectra [9]. Significant improvements have been made in sialolithiasis therapy over the previous two to three decades. The prevalence of gland resection has fallen from 40-50% to less than 7% thanks to the implementation of novel, minimally invasive, and gland-preserving treatments. The conventional approach used to eliminate stones was through a procedure called intraoral submandibular sialolithotomy. The current treatment plan uses a variety of methods, including enhanced and expanded transoral duct surgery (TDS), extracorporeal shock wave lithotripsy (ESWL), and diagnostic and interventional sialendoscopy (intSE). The combination of several treatment methods has been shown to increase the efficacy of therapy, especially when performed endoscopically and transcutaneously [10].

In the past decade, notable progress has been made in the field of intraductal shock-wave lithotripsy (ISWL). The method, which initially produced unsatisfactory results, has been substantially improved through the introduction of innovative instruments, equipment, materials, and techniques [11]. Integral approach techniques have been improved and modernized, with notable updates in the procedures for TDS. These advancements include the adoption of sialendoscopy-assisted TDS for submandibular stones and the incorporation of a retropapillary channel for distal parotid sialolithiasis. The ongoing developments in this area suggest that therapeutic procedures for the primary salivary glands may undergo significant changes. In particular, ISWL has shown promising results and has partially supplanted ESWL and TDS in the submandibular gland. It is imperative to stay current with these advancements to ensure optimal patient care [12]. Untreated cases of sialolithiasis may lead to submandibular lymphadenomegaly and non-odontogenic neck abscess [13,14]. Finally, the main objective of treating sialolithiasis should be to maintain gland function while exposing the patient to minimal risk and pain.

## Conclusions

Sialoliths should be investigated for facial and submandibular pain, especially during meals. Appropriate imaging modalities and comprehensive history should be used to confirm the diagnosis and pinpoint the spot of calcification. Sialogogues, non-steroidal anti-inflammatory drugs (NSAIDs), and antibiotics are effective treatments for most cases of sialolithiasis with a good prognosis. Minimally invasive treatments have higher success rates and lower morbidity than conventional surgery. Sialadenectomy for sialolithiasis is rarely necessary with modern treatment methods.
